# Quantitative fit-test concordance of a pair of similar-fit 3M Aura respirator models, 3M 9320A+ and 3M 1870+: A randomized crossover study

**DOI:** 10.1017/ice.2022.67

**Published:** 2023-02

**Authors:** Daryl Lindsay Williams, Benjamin Kave, Fiona Begg, Charles Bodas, Irene Ng

**Affiliations:** 1 Department of Anaesthesia and Pain Management, Royal Melbourne Hospital, Parkville, Australia; 2 University of Melbourne, Parkville, Victoria, Australia; 3 Respiratory Protection Program, Royal Melbourne Hospital, Parkville, Australia

## Abstract

**Objective:**

Some manufacturers provide information on similar-fit model pairings of filtering facepiece respirators (FFRs), suggesting that fit-test outcome of one model helps predict the other. This guidance may be useful during crisis capacity when FFR supplies and/or fit tests are constrained. The purpose of this study was to compare quantitative fit-test (QNFT) results and concordance between a pair of similar-fit 3M Aura FFRs: the 3M 9320A+ and 3M 1870+.

**Methods:**

All participants completed online training and a QNFT with both respirators. The order of the respirator being examined first was randomly allocated. The outcomes included QNFT pass rate, concordance between the 2 models, overall and individual fit factors, and percentage of male and female participants who passed or failed the QNFT.

**Results:**

We recruited 1,000 participants (668 females and 332 males). The QNFT pass rate, overall fit factors, and individual fit factors were significantly higher for the 3M 9320A+ than the 3M 1870+ FFR. The concordance between the models was “fair” (κ coefficient, 0.38). Male participants who passed a QNFT with either of the FFRs had 96% chance of passing the QNFT for the alternate model. Female participants who passed the 3M 1870+ had 97% chance of passing the QNFT for the 3M 9320A+ model. However, ∼1 in 12 females who passed the QNFT for the 3M 9320A+ failed the QNFT for the 3M 1870+ FFR.

**Conclusions:**

Similar-fit paired FFR models may provide a consequentially different level of respiratory protection, especially for women. Our findings are important for FFR stockpiling and fit-testing strategies, especially during crisis capacity.

Filtering facepiece respirators (FFRs) are the most common form of respiratory protective equipment (RPE) used by healthcare workers (HCWs) to protect themselves from airborne infectious pathogens.^1^ HCWs should be fit tested as part of an integrated respiratory protection program (RPP) to ensure that the make and model of respirator achieves an adequate fit.^2–4^

The Centers for Disease Control and Prevention (CDC) describes strategies to optimize personal protective equipment (PPE) including RPE during conventional, contingency and crisis capacity.^5^ During periods of anticipated shortages of N95 and P2 respirators, annual fit testing may be temporarily suspended, just-in-time fit testing may be utilized, and limited reuse of FFRs may be considered.^6,7^ In addition, manufacturers may provide guidance for nearly identical models of FFRs (ie, pairs), suggesting that the fit-test outcome of one model can help predict fit test outcome of the other.^8^ This information may be of assistance during contingency or crisis capacity, when one of the fit-tested respirator pairs becomes unavailable and/or when fit-testing supplies and services are constrained.

Currently, high-quality evidence supporting the concordance of quantitative fit-test (QNFT) pass rates between similar-fit models produced by the same manufacturer is lacking. Although many models appear to be identical in shape and size, there may be minor differences in manufacturing processes and/or sourcing of raw materials across jurisdictions, which may result in subtle structural differences. The multilayer construction of the respirator may also be different between the paired models, depending on the need for surgical-level fluid protection.^9,10^ All of these elements can potentially alter the QNFT pass rates.

Along with several other studies, our RPP data have demonstrated that 3-panel flat-fold FFRs, such as the 3M Aura, produced high QNFT pass rates, provided high levels of comfort and, therefore, have been the commonly preferred FFRs by HCWs.^11–14^ The purpose of this study was to compare the concordance of QNFT results between 2 models of the 3M Aura FFRs: the 3M 9320A+ and the 3M 1870+ (3M, St. Paul, MN). These 2 models were chosen because they were listed by the manufacturer as one of the similar-fit paired models.^8^ As of November 5, 2021, they were also the most highly stocked 3-panel flat-fold FFRs in our state supply chain.^15^ The results of this study could potentially help guide procurement decisions and better manage surge-capacity scenarios.

## Methods

This prospective randomized crossover study was approved by the Melbourne Health Human Research Ethics Committee (QA no. 2020174). The study was conducted through the Royal Melbourne Hospital Respiratory Protection Program from July 6 to August 10, 2021, during a period of low prevalence of coronavirus disease 2019 (COVID-19), with an average statewide 7-day case number of no more than 20.^16^

As part of the RPP requirement, all of the participants completed online training, according to the manufacturer’s instructions, on the donning, user seal check, and doffing techniques for different types of N95/P2 FFRs, including the two 3M Aura 3-panel flat-fold FFRs used in this study.^17,18^ The order that the 2 respirators were tested first for this study was randomly allocated according to the computer-generated randomization method and stratified into male and female groups Fit testing on the alternate respirator was conducted immediately following the initial fit test. Quantitative fit testing was performed by competent fit-test operators using the ambient aerosol condensation nuclei count method on a Portacount machine (PortaCount Pro+ 8048, TSI, St Paul, MN). The testing was conducted according to the US Occupational Safety and Health Administration filtering facepiece protocols with 4 conventional exercises.^2^ All participants were clean shaven within 24 hours of fit testing and had taken a QNFT with a different brand of 3-panel flat-fold FFR before the study commenced.

The test was observed throughout by a competent operator, and any breach of the protocol was addressed by recommencing the test. Real-time measurements were not allowed during fit testing. If the participant failed their initial fit test, only general guidance that would normally be available to staff in clinical donning zones was allowed. The fit tester reiterated general guidance pertaining to the following procedures: (1) ensuring the mask was open fully with maximal vertical height on the face; (2) ensuring the top strap was over the crown of the head; (3) ensuring the bottom strap was around the neck and below the ears; (4) molding the nasal bar with 2 hands; and (5) performing a positive-pressure fit check and a negative-pressure fit check. Forced fit testing was discouraged, and a maximum of 3 fit-test attempts with general guidance were allowed for each respirator.

The primary outcome was the QNFT pass rate, with passing defined as a harmonic mean overall fit factor of >100, as recommended in the Australian Standards for half-face P2 respirators including FFRs.^4^ Secondary outcomes included the concordance of passing the QNFT between the 2 Aura FFR models, overall fit factors, individual fit factors for each exercise, and the percentage of participants who passed and/or failed both FFRs. Subanalyses were performed to compare the QNFT results between male and female participants, including the overall pass rate; overall fit factor; and the proportion of participants who passed or failed QNFTs with both FFRs or who passed one but failed the QNFT with the alternative model.

### Statistical analysis

The QNFT pass rate with the 3M 9320A+ was ∼96% based on our RPP data and a previous study we conducted,^14^ in which 1,876 QNFTs were passed among 1,946 QNFTs performed by our HCWs. To demonstrate a clinically important difference of up to 3%, at least 971 participants per group would be required for a power of 0.8. Therefore, we aimed to include data from 1,000 consecutive participants of the RPP who provided complete data sets. Basic demographic information was collected via the RPP survey using REDCap, hosted by the Royal Melbourne Hospital. The QNFT results were transferred directly from the Portacount machine onto the RPP REDCap database.

Descriptive statistics have been used to present the demographic data, QNFT pass rates, and quantitative fit factors. The McNemar test and the Wilcoxon signed-rank test were used to compare the pass rates and fit factors, respectively, between the two 3M Aura FFR models. The χ^2^ test and the Wilcoxon rank-sum test were used to compare the pass or failure rates and the fit factors, respectively, between female and male participants. A *P* value of <.05 was considered statistically significant. We used κ statistics to examine the agreement of QNFT pass rates between the 3M 9320A+ and the 3M 1870+ FFR models. The κ value was interpreted to indicate consistency between the 2 models as follows: <0.21 was “poor,” 0.21–0.40 was “fair,” 0.41–0.60 was “moderate,” 0.61–0.8 was “good,” and 0.81–1.0 was “very good.”^19^ Statistical analyses were performed using Stata version 13.0 software (Statacorp, College Station, TX).

## Results

In total, 1,000 participants with complete data sets were included in this study: 668 were women and 332 were men. The average body mass index was 25.4±5 kg m^−2^. Overall, 497 participants performed the fit test with the 3M 9320A+ FFR first, and 503 undertook the fit test with the 3M 1870+ FFR first.

The QNFT pass rate with the 3M 9320A+ FFR was 94.6%, compared to the pass rate with the 3M 1870+ FFR of 91.7% (*P* = .001). The overall fit factor and individual fit factors for each of the 4 exercises were significantly higher for the 3M 9320A+ FFR than for the 3M 1870+ FFR (Table 1). The concordance (ie, κ coefficient) of the pass rates between the 2 FFR models was 0.38, which was categorized as “fair.” Overall, 89.2% of the participants passed the QNFTs with both FFRs, and 3.9% failed both.

**Table 1.** Comparison of Quantitative Fit-Test Results Between 3M Aura 9320A+ and 3M Aura 1870+ Respirators

Variable	3M 9320A+ (n=1,000)	3M 1870+ (n=1,000)	*P* Value
Passed fit test, no. (%)	946 (94.6)	917 (91.7)	.001*
Overall fit factor, mean (SD)	183 (37.9)	175.0 (45.4)	<.001*
**Individual fit factor, mean (SD)**			
Bending over	186.0 (38.4)	179.0 (47.6)	<.001*
Talking	184.4 (40.6)	177.8 (47.7)	<.001*
Head side to side	187.4 (40.9)	181.1 (49.9)	<.001*
Head up and down	181.9 (48.4)	173.3 (57.2)	<.001*

Note. SD, standard deviation.

*Statistically significant.

Male participants were more likely than female participants to pass the QNFT with the 3M 9320A+ FFR (relative risk [RR], 1.03; 95% confidence interval [CI], 1.005–1.06) and the QNFT with the 3M 1870+ FFR (RR, 1.10; 95% CI, 1.06–1.13). Both the QNFT pass rates and the overall fit factors were significantly higher among male participants than female participants for both FFR models (Table 2). A significantly higher proportion of male than female participants passed the QNFTs with both FFRs, and more female participants failed the QNFTs with both FFRs than male participants (Table 2).

**Table 2.** Comparison of Quantitative Fit-Test Results Between Male and Female Participants, With Both 3M Aura 9320A+ and 3M Aura 1870+ Respirators^a^

Variable	Male Participants (n=332)	Female Participants (n=668)	*P* Value
3M Aura 9320A+			
• Passed fit test, no. (%)	321 (96.7)	625 (93.6)	.04*
• Overall fit factor, mean (SD)	187.2 (32.2)	181.0 (40.3)	.006*
3M Aura 1870+			
• Passed fit test, no. (%)	324 (97.6)	593 (88.8)	<.001*
• Overall fit factor, mean (SD)	185.2 (31.5)	170.0 (50.2)	<.001*
Passed fit test for both 3M Aura respirators, no. (%)	316 (95.2)	576 (86.2)	<.001*
Failed fit test for both 3M Aura respirators, no. (%)	3 (0.9)	26 (3.9)	.008*
Passed 3M 9320A+ but failed 3M 1870+, no. (%)	5 (1.5)	49 (7.3)	<.001*
Passed 3M 1870+ but failed 3M 9320A+, no. (%)	8 (2.4)	17 (2.5)	.9

Note. SD, standard deviation.

a For pass/fail entries, values are expressed as absolute number (percentage). For fit factor entries, values are expressed as mean overall fit factor (SD). In either case, the *P* value refers to the comparison between results of male and female participants.

*Statistically significant.

A relatively small and similar proportion of male and female participants passed the QNFT with the 3M 1870+ but failed the QNFT with the 3M 9320A+ (2.4% vs 2.5%; *P* = .90) (Table 2). On the contrary, a significantly larger proportion of female than male participants passed the QNFT with the 3M 9320A+ but failed the QNFT with the 3M 1870+ (7.3% vs 1.5%; *P* < .001).

## Discussion

Our study is the first hospital-based investigation to compare the concordance of QNFT results between a pair of commonly used and similar-fit 3M Aura FFR models, the 3M 9320A+ and the 3M 1870+. Significantly, we have demonstrated that a higher proportion of female than male participants failed to pass the QNFT with the 3M 1870+ FFR model, despite passing the QNFT with the 3M 9320A+ FFR model (7.3% vs 1.5%; *P* < .001). This finding has important implications for FFR stockpiling and fit-testing strategies, especially during contingency or crisis capacity.

The two 3M Aura FFR models have almost identical shape and size in their design. However, a closer examination revealed some subtle differences that may account for the different QNFT pass rates and only fair agreement between the 2 models. Compared to the 3M 9320A+ FFR, the 3M 1870+ FFR has a thicker and wider nasal foam and a firmer nasal bar, which could be difficult to mold to achieve a seal. It also has more tension in the straps (as shown in our experiment in Appendix 1 online), which may disrupt the seal of a nonrigid 3M Aura in some participants. The significant differences between male and female participants observed in this study could be due to their anthropometric differences. This finding is consistent with previous research,^20,21^ which demonstrated that increasing facial length, commonly found in males, improved respirator fit.

Under conventional circumstances, staff should be fit tested in an established RPP with each respirator model they use, as recommended by international and local standards.^3,4^ Health service procurement should not operate under the assumption that paired models produced by the same manufacturer provide the same level of fit and, therefore, substitute one model for the other because this can lead to a lack of protection for a portion of the workforce. For example, in this study, ∼1 in 12 female participants who passed the QNFT with the 3M Aura 9320A+ FFR did not pass the QNFT with the 3M 1870+ FFR.

On the other hand, during contingency or crisis stages of a pandemic, a risk-based approach could potentially be adopted instead, with consideration given to the hierarchy of controls and local regulatory agency advice in place at the time. For example, it could perhaps be considered acceptable for men, who were shown to have 96% chance of passing QNFT for the alternate 3M Aura model to wear the alternate respirator in low-risk settings, when fit testing is constrained or FFRs are short supply. However, such decisions would need to be ratified by relevant authorities consistent with contemporaneous regulatory advice. High residual respiratory biohazard risk should dictate that staff undertake just-in-time fit testing to ensure high levels of respiratory protection as the last layer of HCW defense.^22^ Knowing the QNFT results of paired respirators would help to streamline the just-in-time fit testing process.

This study had several limitations. First, participants undertook their tests consecutively on the 2 models, that is, they took the second test immediately after the first test. This procedure may have conferred a training effect for the second model tested. We randomized the first FFR tested to prevent this potential effect. Second, the fit tester could not be blinded to the model of FFR being tested due to the need to observe the participant. Third, some of the difference could potentially be due to Portacount repeatability. However, we limited any variation by using standardized operating procedures and by randomizing the respirator fit-test order. Lastly, we have only studied 1 ‘pair’ of similar-fit FFR models. Many similar pairs exist, and we recommend further research to determine concordance between these paired similar models. Nevertheless, there is a reasonable likelihood that the conclusions of this study are generalizable to other FFR pairs.

In conclusion, we demonstrated that similar-fit paired FFR models may provide a consequentially different level of respiratory protection for some individuals, particularly women. Even minor modifications in the manufacturing of near identical P2/N95 respirators may result in significant changes in QNFT pass rates. Our study findings are important to procurement departments, RPP administrators, and health services in their FFR stockpiling and fit-testing strategies, especially during times of contingency and crisis surge capacity.

## Data sharing statement

All the individual deidentified data that support the findings of this study are available upon request from the corresponding author. Study protocol and statistical analysis are also available. Information will be available immediately following publication until 5 years after publication.

## Appendix 1. Strap elasticity study of 3M Aura 9320A+ versus 3M Aura 1870+

Material properties such as elastic modulus can be determined empirically to describe a material’s elasticity (ie, its resistance to elastic deformation). However, this modulus is a physical property of a given material (eg, an elastic material 3 mm thick will have the same modulus of elasticity as a 1-mm-thick portion of the same material but will require greater force to stretch the same distance).

The thickness of the strap is therefore an important contributor to user-experienced stretch resistance. As such, the force required to stretch different straps by a set distance is a simple, objective metric that enables comparison. To compare each strap’s stretch resistance, the force required to stretch a portion of strap from 60 mm to 80 mm (ie, 33%) was determined using a force gauge. On average, the 3M 1870+ straps required 22% more force than the 3M 9320A+ straps to be stretched an equivalent distance.

**Table:**

Force required to stretch strap by 33% (N)	
3M 9320A+	3M 1870+
0.9	1.1
0.9	1.2
0.9	1.1
0.9	1.1
0.9	1
0.9	1.1
Mean	
0.9	1.1
Relative % Difference	
…	22%
